# IDH2-mutated near ETP-ALL with aggressive leukemia cutis and brisk response to venetoclax and decitabine

**DOI:** 10.1016/j.lrr.2023.100408

**Published:** 2023-12-30

**Authors:** Poorva Vaidya, Huan-You Wang, Michelle D. Don, Brian R. Hinds, James K. Mangan

**Affiliations:** a Department of Internal Medicine, Division of Hematology-Oncology, Moores Cancer Center, University of California, San Diego, La Jolla, CA, United States; b Department of Pathology, University of California, San Diego, La Jolla, CA, United States; c Department of Dermatology, University of California, San Diego, La Jolla, CA, United States; d Department of Internal Medicine, Division of Blood and Marrow Transplantation, Moores Cancer Center, University of California, San Diego, La Jolla, CA, United States

**Keywords:** T-cell lymphoblastic leukemia, Leukemia cutis, Venetoclax, Hypomethylating agents

## Abstract

Near early T-cell precursor acute lymphoblastic leukemia (ETP-ALL) is a rare hematologic malignancy, for which second line therapeutic options are limited. T-cell leukemias are also rarely associated with leukemia cutis, which is more often seen in leukemias of myeloid origin. We present the case of an adult male diagnosed with near ETP-ALL, with IDH2 and DNMT3A mutations, suggestive of a myeloid origin, and leukemia cutis. After the patient progressed on hyper-CVAD and nelarabine, we treated him with the BCL-2 inhibitor venetoclax and the hypomethylating agent decitabine. The regimen induced a rapid bone marrow response and resolution of the leukemia cutis.

## 1 Introduction

Early T-cell precursor acute lymphoblastic leukemia (ETP-ALL) is a rare hematologic malignancy in children and adulthood, and near-ETP-ALL is even rarer [1]. First-line treatment of T-ALL in adults involves intensive, multi-agent chemotherapy [2]. Younger adults can be treated with pediatric-inspired protocols, whereas asparaginase therapy is attenuated or absent in protocols used for older adults. Unfortunately, therapeutic options for relapsed or recurrent T-ALL are sparse [3]. In the second line setting, the T-cell directed purine analog, nelarabine, is approved for use as monotherapy and has been shown to confer a complete remission rate of 36 % and an overall response rate of 41 % [4,5]. More recently, the BCL-2 inhibitor venetoclax (VEN) and hypomethylating agents (HMA), which are approved for frontline treatment of acute myeloid leukemia (AML) in the elderly, have been studied in the treatment of relapsed T-ALL [6], [7], [8]. The combination of venetoclax and the bcl-XL inhibitor navitoclax with chemotherapy has shown potential in a phase I study of 47 patients with relapsed/refractory ALL that included a substantial T-ALL population [9]. In addition, the combination of venetoclax with the proteasome inhibitor bortezomib has also demonstrated activity in a small case series [10]. It remains to be determined which patients with T-ALL are the best candidates for therapy with VEN and HMA, and whether certain T-ALL characteristics predict more robust response to this regimen.

Even sparser data exist on the diagnosis and management of cutaneous manifestations of T-ALL. Leukemia cutis is well described in acute myeloid leukemias; however, incidence in both T- and B-cell ALL and lymphomas is exceedingly rare or underreported [11,12]. There are no comprehensive guidelines regarding the optimal management of leukemia cutis in T-ALL, especially when it manifests after first line therapy.

We present the case of an adult male patient with relapsed near ETP-ALL with leukemia cutis and genomic mutations suggestive of myeloid origin, who responded to third line therapy with VEN and decitabine with complete resolution of cutaneous disease. To our knowledge, this is the first case of T-ALL associated leukemia cutis reported to have complete response to treatment with VEN/HMA. The remarkable cutaneous response was also accompanied by a deep bone marrow response, with reduction of T-lymphoblasts from 85–90 % to 5–10 % by immunohistochemistry after a single cycle of VEN/HMA.

## 2 Case

A 71 year-old male with past medical history of hypertension, type 2 diabetes, benign prostatic hyperplasia and hypothyroidism presented to our hospital's bone marrow transplant service with a two-month history of 25-pound unintentional weight loss, night sweats and lymphadenopathy. He was found to have cytopenias with WBC 2100/ul (17 % neutrophils, 63 % lymphocytes, 18 % blasts), hemoglobin 8.1 and platelets 177,000/ul. Bone marrow biopsy was performed and flow cytometric analysis showed blasts comprising 52 % of total leukocytes, expressing cytoplasmic CD3 (subset), CD5 (more than 75 % of blasts), CD7 (bright), CD34, and TdT (subset) with co-expression of CD13, but negative for CD117 or cMPO, most consistent with a near early T-cell precursor acute lymphoblastic leukemia (near ETP-ALL). When confronted with a suspected case of ETP-ALL, the differential diagnosis includes near ETP-ALL and also T/myeloid mixed phenotype acute leukemia. In our case, near ETP-ALL is favored over ETP-ALL because of the high frequency (>75 %) of blasts expressing CD5 [13]. The distinction between ETP-ALL and T/myeloid mixed phenotype acute leukemia (MPAL) can be problematic because the expression of myeloid markers is characteristic of ETP-ALL, but MPO positivity is required to define myeloid lineage and MPO was negative in our case, thus excluding T/myeloid MPAL [14]. Interestingly, there were normal cytogenetics, but next generation sequencing (NGS) showed mutations in IDH2 (R140Q), KRAS (G12V), NOTCH1 (F1592S) and DNMT3A (Q356*) with variable allele frequency of 38 %, 36 %, 5 %, and 75 % respectively. Of note, the allele frequency of the NOTCH1 mutation in this case is quite low and NOTCH1 mutations are actually quite rare in ETP-ALL, though very common in other subtypes of T-ALL [15], so it is unlikely to be an important driver mutation in our case. Magnetic resonance imaging (MRI) of the brain showed diffuse pachymeningeal involvement and flow cytometry of cerebrospinal fluid (CSF) was consistent with near ETP-ALL. He subsequently completed hyper-CVAD through cycle 2B. Post-treatment bone marrow biopsy showed minimal residual disease with 5 % blasts. CSF showed CNS clearance after intrathecal chemotherapy treatments. He was then started on second line treatment with nelarabine monotherapy.

**Fig. 1:**
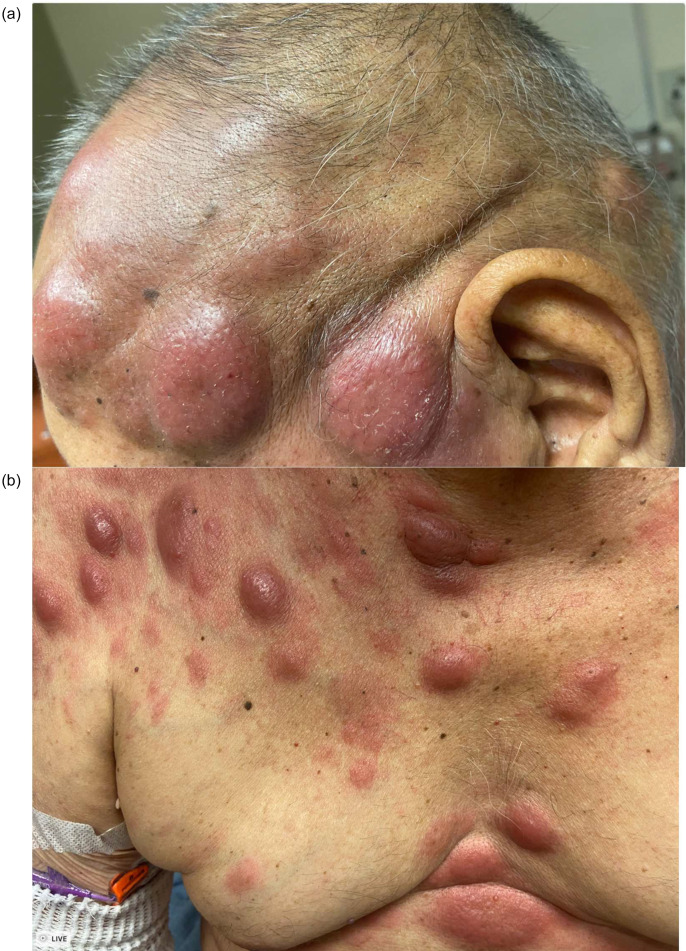
Panels (A) and (B) depict the patient's T-cell lymphoblastic leukemia cutis at the time of disease progression.

**Fig. 2:**
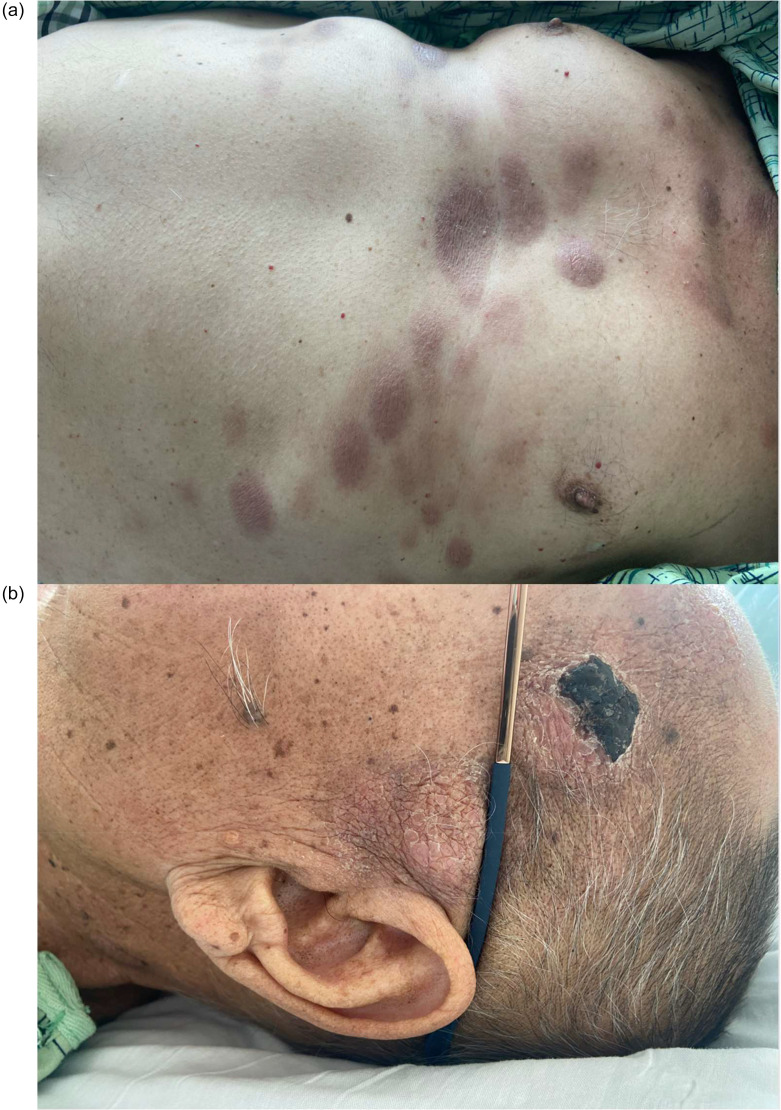
Panels (A) and (B) depict the patient's skin lesions following treatment with decitabine and venetoclax.

At the start of cycle 1 of nelarabine, the patient reported new, hive-like lesions on his face and scalp that initially mildly improved with ongoing nelarabine, but then continued to progress to his face, back and extremities (Fig. 1(A) and (B)). Punch biopsy of a 1.5 cm lesion of the upper back was performed. Histopathologic examination revealed sheets of large atypical lymphoblasts positive for TdT and cytoplasmic CD3 with preservation of CD5 and CD7. Concurrently, on post-nelarabine bone marrow biopsy, the patient was found to have full-blown near-ETP-ALL with up to 85–90 % T-lymphoblasts. At this juncture, he was started on VEN and the HMA decitabine. By day 6 of treatment, lesions were nearly resolved, simply reduced to flat, violaceous discoloration (Fig. 2(A) and (B)). He tolerated treatment with no toxicities. Bone marrow biopsy showed a dramatic marrow response, with only 5–10 % residual T-lymphoblasts at the end of one cycle of therapy. The patient continued on VEN/HMA with a complete cutaneous response and ongoing partial bone marrow response before progressing and being enrolled in a clinical trial.

## 3 Discussion

Relapsed/refractory T-ALL remains a challenging disease with limited treatment options. It is even rarer for T-ALL to be associated with leukemia cutis, a phenomenon much more often associated with leukemias of myeloid origin. However, our patient with near-ETP-ALL with leukemia cutis had a brisk, complete response to VEN/HMA—a third line treatment for his T-ALL, but a frontline treatment for patients diagnosed with AML. In reflecting on his clinical presentation and robust response to VEN/HMA, it is important to consider the mutations identified in this patient (IDH2 and DNMT3A) that are more typically associated with myeloid neoplasms and/or MPAL [16].

Clonal hematopoiesis of indeterminate potential (CHIP) is a subset of somatic genomic alterations without evidence of cytopenias, dysplasia, or an overt hematologic malignancy. Until recently, it was thought that CHIP was exclusively implicated in myeloid malignancies, while mosaic chromosomal alterations (mCAs) were implicated in lymphoid malignancies. In 2021, Niroula et al. published an analysis of over 55,000 patients which identified distinct lymphoid clonal hematopoiesis (L-CH) also associated with increased risk of lymphoid malignancies [17]. The presence of clonal hematopoiesis in the lymphoid setting was further reported in a study of 400 adult patients with ALL [18]. The mutations identified in our patient, DNMT3A and IDH, were identified in five percent and two percent of ALL cases, respectively. These data support that precursor clonal hematopoiesis lesions can propagate lymphoid malignancies and this mechanism of leukemogenesis likely played a role in our patient's presentation.

Future directions include prognosticating lymphoid malignancies by their myeloid CHIP precursors and establishing appropriate subsequent line treatment protocols. In our case of IDH and DNMT3A mutated near ETP-ALL, the response to VEN/HMA was rapid and sustained, and the excellent response seen in our patient with near ETP-ALL is typical for patients with IDH1/2 mutations in AML [19]. The patient described here also had a DNMT3A mutation which is seen in over 20 % of patients with AML, and it is possible that the DNMT3A mutation may also confer responsiveness to VEN/HMA in our case, though the presence of a DNMT3A mutation in AML is not associated with a superior response to VEN/HMA as is the case with IDH1/2 mutations [20]. However, it remains unclear whether this response would be expected in all T-ALL cases and which patients would be optimal candidates for similar treatment.

As previously mentioned, leukemia cutis is exceedingly rare and thus not well-studied in T-ALL. Moreover, as data on CH and myeloid mutations in lymphoid malignancies is just recently emerging, there is no evidence to support a clear association between the development of cutaneous lesions in T-ALL and pre-existing CHIP. It has been previously reported that a case of Sweet's Syndrome (SS), a neutrophilic dermatosis often found in hematologic malignancies, which occurred in a patient with CHIP responded to the HMA 5-azacitidine [21]. Although SS is pathologically distinct from leukemia cutis, it is of interest to determine if dermatologic manifestations associated underlying CH are more responsive to HMA-based regimens. If this relationship were to be established, then future similar patients with T-ALL and leukemia cutis could benefit from therapies which include HMAs, and the presence of an underlying IDH mutation could direct the addition of VEN to the HMA, given the outstanding responses seen in myeloid malignancies with IDH1/2 mutations treated with VEN/HMA.

## 4 Conclusion

The use of the Bcl-2 inhibitor VEN in combination with the HMA decitabine may be effective as second line treatment in relapsed/refractory T-ALL with IDH mutations, and in our patient, this therapy also demonstrated dramatic and rapid efficacy in the treatment of aggressive, highly proliferative leukemia cutis.

## Informed consent

This article is a case report that uses photographs of the subject of the case report that are completely de-identified. There is no information in the text or in the photos that can result in the identification of the patient. Therefore, informed consent was not required per our institutional IRB policies.

## CRediT authorship contribution statement

**Poorva Vaidya:** Conceptualization, Writing – original draft, Writing – review & editing. **Huan-You Wang:** Conceptualization, Writing – review & editing. **Michelle D. Don:** Conceptualization, Writing – review & editing. **Brian R. Hinds:** Conceptualization, Writing – review & editing. **James K. Mangan:** Conceptualization, Writing – review & editing, Writing – original draft.

## Declaration of Competing Interest

The authors declare that they have no conflict of interest.
